# The Effect of a Quality Improvement Project on Improving Patients’ Willingness to Receive an Influenza Vaccination in the Emergency Department

**DOI:** 10.1007/s10903-023-01574-2

**Published:** 2024-01-04

**Authors:** Paola H. German, Mark Lazenby, Susanne Phillips, Angela Jun

**Affiliations:** Sue and Bill Gross School of Nursing, University of California Irvine, 854 Health Sciences Road, Irvine, CA 92697 USA

**Keywords:** Influenza vaccine, Immunization, Vaccination campaign, Emergency department, Immunization education

## Abstract

The aim of this project was to increase willingness to receive the influenza vaccine to the optimal rate of ≥ 70%. Low acuity adult patients who visited an Emergency Department (ED) were assessed regarding their willingness to receive the influenza vaccine before and after an educational intervention that included a provider recommendation and an educational handout. A total of seventy-six patients (n = 76) were assessed. Patients’ willingness to receive the influenza vaccine rose from 29% pre-intervention to 72% post-intervention without disrupting the clinical flow in a busy ED. Similar vaccine educational strategies can be applied to influenza and other vaccines in EDs  to increase vaccination willingness in patients, including those who use the ED as a primary point of contact for healthcare, decreasing the burden of influenza illness in the community.

## Background

According to the last known data, in 2015, the economic burden of influenza illness on the U.S. healthcare system and society was approximately $11.2 billion [1]. During the 2019–2020 influenza season, the Center for Disease Control and Prevention (CDC) estimated approximately 39–56 million influenza illnesses led to 410,000–740,000 hospitalizations and 24,000–62,000 deaths [2]. Many of these hospitalizations and deaths may have been prevented with the influenza vaccine, which the CDC proposes as the most efficient way to prevent risks associated with influenza-related disease [3].

During the 2020–2021 influenza season, influenza vaccination rates in the U.S. increased from 48.4% to 52.1% [3]. However, this still did not meet the optimal rate of 70% established by Healthy People 2030 [4], a national effort that sets objectives for improving the health of the people living in the U.S. The Advisory Committee on Immunization Practices suggests that the greatest barrier to achieving target vaccination rates is limited awareness of vaccines among adult patients and providers [5]. Additional studies also highlight lack of awareness, misperceptions, and limited access to vaccinations as contributors to the underachievement of ideal influenza vaccination rates [6–8].

The need to overcome vaccination barriers and facilitate vaccine access to underserved communities and high-risk patients has been highlighted by the recent COVID-19 pandemic [3]. Emergency Department (ED) visits offer a unique opportunity to overcome these barriers by providing patients with vaccine education and recommendation [7–11]. ED services are used by the general population, including people with public health insurance and the uninsured, who widely use the ED as the only access point for health care needs [12, 13]. Approximately 90% of ED patients under 65 years of age are discharged home the same day [12], many presenting with less urgent to non-urgent complaints. Although EDs have been providing vaccines such as tetanus [14] and hepatitis A [15] as prevention for years, efficient strategies regarding education and administration of influenza vaccines in the ED are missing [9, 11, 16–18]. The ED provides a unique opportunity to reach out to the general population, including underserved patients who use the ED.

Through an easy-to-implement, cost-effective vaccination education strategy, this quality improvement project's purpose was to increase the influenza vaccination willingness of patients who use the ED, including those who use it as an access point for healthcare needs and who otherwise have limited or no access to vaccines or vaccine education.

## Methods

This quality improvement project employed a pre-post single-arm design to assess whether an easy-to-implement educational intervention improved the willingness of patients to receive an influenza vaccine during their visit to the ED.

### Setting/Participants

The project was implemented at a Level I trauma center and academic ED in Orange County (OC), California. In OC, 29.6% of the population are foreign-born, while 34.1% are Hispanic or Latino, and 22.8% are Asian [19]. Furthermore, 24.9% of the population are covered by Medi-Cal, California’s Medicaid program, 9.1% by Medicare, while 7.5% of persons under 65 years of age have no health insurance. In a period of 12 months, 1 in 5 residents visited an ED, with patients between 18 and 64 years of age visiting at higher rates than other groups [20].

Participants included low-acuity patients who presented to the ED with Emergency Severity Index (ESI) IV—less urgent—and ESI V—non-urgent—and were seen at the fast-track area by a nurse practitioner (NP). Participants were over 18 years old and Spanish or English speakers who had not received an influenza vaccine. Exclusions included (1) patients identified as ESI I—life-threatening, ESI II—high risk, or ESI III—stable; (2) patients with altered mental status; and (3) patients with severe allergies to any ingredient in a flu vaccine or medical issues such as history of Guillain–Barré Syndrome.

### Ethical Considerations

The project protocol was reviewed by the academic center’s Institutional Review Board (IRB). The IRB determined that the activities did not constitute human subject research or a clinical investigation. Therefore, consent to participate was not necessary.

### Procedures

Between January 9th and February 5th, 2022, a convenience sample of fast-track patients were identified through the electronic health record as they checked into the ED and were asked in triage by the triage registered nurse about their influenza vaccination status. If unvaccinated, the triage nurse asked about their willingness to receive an influenza vaccine if it was offered in the ED.

### Data Collection Timepoint 1

The following data were collected at timepoint 1 by the triage nurse: (1) patients’ willingness to receive a vaccine (“yes” = willing; “no” = unwilling; “not sure” = unsure), and, if they were unwilling or unsure, (2) the stated reasons. Unwilling and unsure patients received the educational intervention.

If the patient was willing to receive a vaccine, (3) demographic characteristics (age in years, sex, race/ethnicity, insurance status) were collected. For unwilling and unsure patients, demographics were collected at timepoint 2 by the Nurse Practitioner (NP) for ED efficiency purposes.

### Educational Intervention

The triage nurse provided the unwilling or unsure patient with an influenza vaccine educational handout “No more Excuses: You need a Flu vaccine” retrieved from the CDC *2021 Flu Vaccination Campaign* online at no cost [21]. This handout was aimed at the general public and addressed facts regarding the influenza vaccine such as why everyone needs a vaccine, how often, vaccine safety, and common vaccine side effects. It was available to patients in Spanish and English. The purpose of the handout was for patients to have the opportunity to read it while waiting to be seen and to serve as a reminder to providers to recommend the vaccine [22, 23]. After the triage process, patients with the handout waited in the lobby to be medically evaluated by an NP. NPs serve as the healthcare providers in the fast-track area of the ED.

During the evaluation, the NPs who treated the patients with the handout provided a recommendation for the vaccine [23–25], answered patients’ questions, and assessed whether the patients read the educational handout (yes/no). NPs were educated beforehand about how to make a strong influenza vaccination recommendation based on methodology provided by the CDC [11, 16].

### Training of NPs Interventionist

An email that included clinical staff educational slides from the CDC Flight Flu 2021 campaign [26] and a PowerPoint presentation of the project was sent to all NPs prior to implementation of the project. The PowerPoint presentation included the background and purpose of the project, evidence-based recommendations, methods, and a process map. The clinical staff educational slides included AICP recommendations, how to address questions, vaccine refusals and perceived barriers, tips on how to make a strong vaccine recommendation, and application of the SHARE model (a five-part approach to make a strong influenza vaccine recommendation: Share, Highlight, Address, Remind, Explain) [26]. In addition, two NPs were selected as “Project Champions” [10] with in-person education regarding the project and recommendation strategy. All NPs were suggested to use the verbiage “you are due for a flu vaccine” [26] when evaluating the patient. Laminated cards, including verbiage and the SHARE methodology, were displayed at the NP workstations as reminders. The primary investigator visited the implementation site multiple times before and during NP shifts to educate them, answer questions, and receive feedback. Intervention fidelity was assessed by whether the NP gave a recommendation to the patient and whether the patient read the handout.

### Data Collection Timepoint 2

After the educational intervention, the NPs reassessed patients’ willingness to receive the vaccine (“yes” = willing; “no” = unwilling; “maybe later” = unsure) and collected patients’ demographic data (timepoint 2).

### Measures

A tracking sheet was developed for triage nurses and fast-track NPs to record data. Data were collected at timepoints 1 and 2, depending on patients’ willingness to receive an influenza vaccine pre-and post-intervention. One tracking sheet was assigned per patient. After the triage nurse collected the data on the tracking sheet at timepoint 1, the tracking sheet was handed to the fast-track NP seeing the patient. The tracking sheets also collected patients’ demographic characteristics.

The original intervention procedures called for vaccine administration at timepoints 1 and 2 among participants who indicated they were willing. Whether participants received the vaccine was the original quality improvement project outcome. However, due to the strain caused by the COVID-19 pandemic on ED resources, actual administration of the influenza vaccine was unfeasible. Thus, willingness to receive the influenza vaccine alone was collected as the intervention outcome. After project completion, the primary investigator debriefed with ED staff concerning the intervention’s effect on ED workflow.

### Analysis

Data from the tracking sheets were entered into a Microsoft Excel spreadsheet by the primary investigator and checked for accuracy by a project team member. The primary investigator organized and analyzed the data in the spreadsheet. Demographic data, changes in willingness by race and ethnicity, and stated reasons for not receiving the influenza vaccine pre-intervention were described using frequencies and percentages and, for age, by measures of central tendency and dispersion.

Participants’ willingness to receive vaccination was described using frequencies and percentages at timepoint 1 (pre-intervention) and timepoint 2 (post-intervention). As this was not a hypothesis-testing intervention, to describe the interventions’ effect on changing participants’ willingness, we compared willingness before and after the intervention. The post-intervention expected results were calculated by multiplying the data collection timepoint 2 frequencies of the “unwilling” and “unsure” by the data collection timepoint 1 percentages of “unwilling” and “unsure” and compared the results to the data collection timepoint 2 actual results.

## Results

Seventy-six unvaccinated participants were included in the project. Their mean age was 43 (± 12.6) years. Forty-two participants (55%) identified as female and 51 participants (67%) as of Hispanic origin. Fifty (66%) participants reported government-funded insurance. Participants’ characteristics are described in Table 1.

**Table 1:** Participant characteristics (N = 76)

Characteristic		
Age (in years)	Mean (SD)	43 (± 12.6)
	Range	18–66

		n (%)
Sex	Female	42 (55)
	Male	34 (45)
Race/Ethnicity	White, Hispanic	51(67)
	White, Non-Hispanic	10 (13)
	Asian	10 (13)
	African American	5 (7)
Insurance status	Government funded^a^	50 (66)
	No insurance^b^	18 (24)
	Private	8 (11)

^a^Government funded includes Medi-Cal and Medicare

^b^No Insurance includes Emergency Medi-Cal coverage

Fifteen nurses collected data at Timepoint 1 and provided an educational handout to 54 participants. Six NPs provided recommendations and collected data at timepoint 2 from 54 participants. During triage screening, 22 (29%) participants were willing to receive an influenza vaccine if offered so they did not need further intervention. Table 2 describes participants’ reasons for not receiving the influenza vaccine prior to the ED visit. Forty-five (59%) participants were not willing to receive the influenza vaccination at the time of visit, and 9 (12%) were unsure (Table 2).

**Table 2:** Stated reasons for not receiving the influenza vaccine prior to the ED visit and for declining the influenza vaccine pre-intervention (N = 76)

Stated reasons	Willing participants (n = 22)	Unwilling and unsure participants (n = 54)
	n (%)	n (%)
Misconceptions/lack of education^a^	16 (73)	35 (65)
Financial/lack of resources	5 (23)	8 (15)
Medical concerns/physical limitations^b^	0 (0)	8 (15)
No answer recorded	1 (5)	3 (6)

^a^Misconceptions/lack of education included: participants did not know they needed one, they never became sick with influenza, they felt they were too young or healthy, they became sick after vaccination, they were not interested, or they did not believe in vaccines

^b^Medical concerns/physical limitations included: participants feared needles, felt too sick or in pain at the time of assessment

Of the total participants who received the intervention (n=54), 50 (93%) received the full intervention, and 4 (7%) received the recommendation but not the educational handout. Of those who received the influenza vaccine educational handout, 41 (82%) reported reading the handout before seeing the NP.

After the intervention, willingness to receive influenza vaccination in the ED was reassessed. Of the 45 participants who initially said they were unwilling to receive the influenza vaccine, 25 (56%) agreed to be vaccinated after the intervention. Table 3 describes the results and compares these results with expected results based on pre-intervention percentages. Table 4 describes changes in willingness by race and ethnicity.

**Table 3:** Willingness, unwillingness, unsurety regarding receiving the influenza vaccine after receiving the intervention (N = 54)

Willingness status after intervention	Willingness status intervention			
	Unwilling (n = 45)		Unsure (n = 9)	
	Actual result	Expected result	Actual result	Expected result
	n (%)	n (%)	n (%)	n (%)
Willing	25 (56)	13 (29)	8 (88)	3 (29)
Unwilling	14 (31)	27 (59)	0 (0)	5 (59)
Unsure	6 (13)	5 (12)	1 (12)	1 (12)

This table compares actual results with expected results based on pre-intervention percentages. Compared with expected results, the rate of willingness to receive the vaccine nearly doubled (1.9 times) among participants who were unwilling before the intervention. Among those who were unsure before the intervention, the rate of willingness was 3 times greater after the intervention

**Table 4:** Changes in willingness by race/ethnicity (N = 76)

		Total sample by race/ethnicity (N = 76)			
		White Hispanic (n = 51)	White non-Hispanic (n = 10)	Asian (n = 10)	African American (n = 5)
		n (%)	n (%)	n (%)	n (%)
Pre-intervention	Willing	18 (35)	1 (10)	3 (30)	0 (0)
	Unwilling or unsure	33 (65)	9 (90)	7 (70)	5 (100)
Post-intervention	Willing	24 (73)	3 (33)	4 (57)	2 (40)
	Unwilling or unsure	9 (27)	6 (67)	3 (43)	3 (60)

Patients from the Hispanic population represented 67% (51) of the total project sample. From the 65% (33) initially unwilling or unsure pre-intervention, 73% (24) were willing post-intervention. White Non-Hispanics represented 13% (n = 10) of the total sample, 33% (n = 3) of the initially unwilling/unsure patients were willing to receive a vaccine post-intervention. Asians represented 13% (n = 10) of the total sample. Of the 70% (n = 7) initially unwilling or unsure to receive a vaccine pre-intervention, 57% (n = 4) had a change in willingness post-intervention. African Americans represented 7% (n = 5) of the total sample, with 100% (n = 5) of them unwilling or unsure to receive a vaccine pre-intervention, and a change of willingness of 40% (n = 2) post-intervention

Compared with expected results, the rate of willingness to receive the vaccine nearly doubled (1.9 times) among participants who were unwilling before the intervention when compared with expected results based on pre-intervention percentages. Among those who were unsure before the intervention, the rate of willingness was 3 times greater after the intervention.

**Fig. 1:**
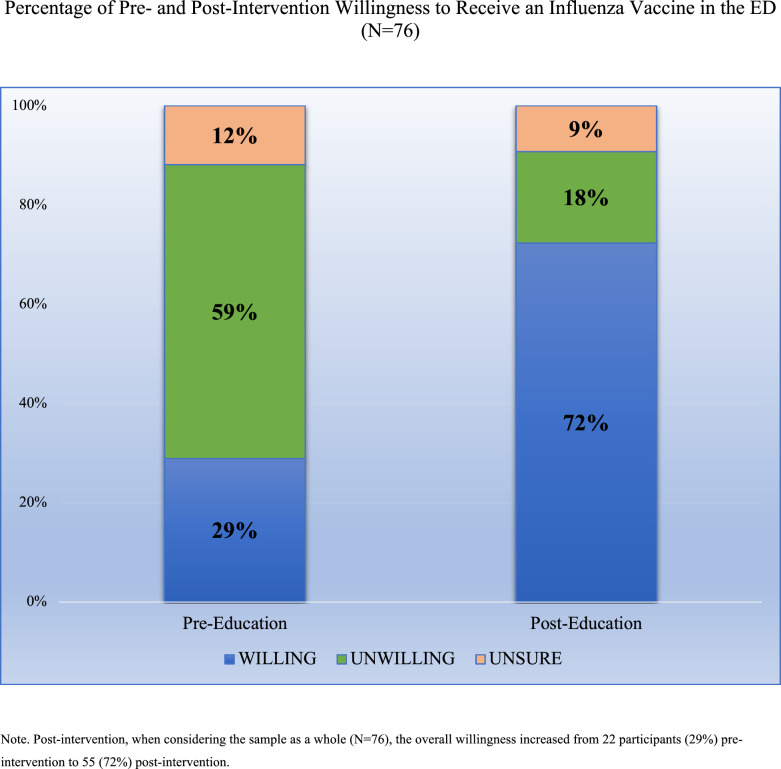
Percentage of pre- and post-intervention willingness to receive an influenza vaccine in the ED (N = 76). *Note* Post-intervention, when considering the sample as a whole (N = 76), the overall willingness increased from 22 participants (29%) preintervention to 55 (72%) post-intervention

Post-intervention, when considering the sample as a whole (N = 76), the overall willingness increased from 22 participants (29%) pre-intervention to 55 (72%) post-intervention (Fig. 1).

## Discussion

This pre-post single-arm quality improvement project assessed whether an easy-to-implement educational intervention improved patients’ willingness to receive the influenza vaccine among a convenience sample of unvaccinated patients who use the ED as an access point for healthcare [7].

This low-cost intervention consisted of an influenza vaccination educational handout and a verbal recommendation by fast-track NPs. The handout was downloaded from the CDC *2021 Flu Vaccination Campaign* webpage, which offers resources and handouts at no cost to providers. The only incurred cost of the intervention was the printing of the handouts. The results, which show an increase in vaccination willingness with a low-cost intervention, could further decrease the financial burden of influenza illness on the U.S. healthcare system and society [1]. Furthermore, the easy-to-implement intervention was embedded within the daily workflow in triage and NPs’ fast-track evaluation of patients to prevent workflow interruption, making this project implementable in busy ED settings, even in times when ED resources were limited [11, 17].

The project results suggest that ED patients will read the educational handout while waiting to be seen by a provider (84%), and that patients can be positively influenced by a provider recommendation. It is suggested that patients are more likely to receive a vaccination from their provider due to their trust in providers as reliable sources of health information [24, 25]. For some patients, the ED is their only access point for healthcare needs, representing the only opportunity of interaction with a provider. The achieved project outcome of 72% willingness to receive an influenza vaccine post-intervention surpassed the optimal rate of 70% established by Healthy People 2030 [4].

Historically, racial disparities exist for influenza vaccination in the U.S., with people who identify as Hispanic and Black/African American having the lowest vaccination rates, and influenza illness unequally affects racial and ethnic minorities and patients with low socioeconomic status [27]. Based on the CDC survey data estimates for the 2021–2022 influenza season, the coverage for influenza vaccines among Hispanic adults was 17.2 percentage points lower (33.8%) than White, non-Hispanic adults (51.0%) [28]. Although non-Hispanic whites represent most of the ED visits in the U.S., blacks and Hispanics report receiving most routine healthcare in the ED and not having a primary care provider [29]. In addition, within the healthcare system, migrants are considered underserved due to lack or limited access to care [30], increasing their dependence on emergency services [31].

The project assessed the participants’ changes in willingness to receive an influenza vaccine by race and ethnicity (Table 4). Patients from the Hispanic population represented the largest portion of the total project sample 67% (n = 51). From the 65% (n = 33) initially unwilling or unsure pre-intervention, 73% (n = 24) were willing post-intervention. In areas where the unvaccinated are represented by minorities and immigrants, the project can assist in increasing vaccination willingness, education, and access. Due to the sensitivity of the data, only participants’ race and ethnicity were collected; immigration status was not. Collection of immigration status by clinicians can create mistrust, lead to stigmatization, and place the patient in a vulnerable place for discrimination and mistreatment [32]. Additionally, it can pose legal risks [33].

Providing the influenza vaccine as the last step in this QI project’s intervention would have been ideal. However, the project was implemented during an unexpected surge of COVID-19 pandemic. Supplies, equipment, and staff resources were shifted to care for a large volume of symptomatic and critical patients. Thus, it was not feasible to provide the vaccine. Although COVID-19 is still present in our society, it is no longer under pandemic status and does not cause the same strain on hospital resources. However, the need to overcome barriers to vaccination is a lesson learned from the COVID-19 pandemic, and action should be taken to prevent surges from happening. Educating patients and creating a culture of vaccination among staff and providers are practices suggested in the literature [11, 16, 17].

Given that the willingness to receive the influenza vaccine in the ED was demonstrated, future hypothesis-testing two-arm intervention trials that compare the intervention with usual care should focus on the feasibility of administering the vaccine in these settings by considering cost justification, financial benefit analyses, and accessible resources. The final result could be to decrease the burden of influenza illness in the ED and the community, therefore, preserving ED resources for higher acuity patients.

### Limitations

The project was implemented in the latter half of the influenza season, which may have decreased the number of eligible project participants due to patients already being vaccinated or not wanting to become vaccinated so late in the season [18]. Some unvaccinated low acuity patients who met inclusion criteria may not have been included due to refusal to participate, data we did not keep. Limited ED resources and overcrowding related to the surge of COVID-19 resulted in frequent closure of the fast-track area, causing a fewer number of patients seen [9]. Participants’ race and ethnicity were collected, but preferred language was not. A relatively small number of nurses and NPs (n = 21) participated in the project, and even if participating nurses and providers thought that the project was feasible and worth expanding to other areas of the ED during the next influenza season, it is not known how others perceived the project. Lastly, this project was not statistically powered.

## New Contributions to Literature

The COVID-19 pandemic highlighted the need to overcome vaccination barriers and facilitate vaccine access to underserved communities and high-risk patients. Some of this responsibility may lie in the ED. This project showed that an easy-to-implement influenza vaccination educational strategy can be successfully utilized in a busy ED to increase the willingness of patients to receive the influenza vaccination. These results are important because EDs serve the general population, including the underserved communities who use the ED as a primary point of access for healthcare, providing a unique opportunity to educate and potentially vaccinate populations with limited or no access to vaccine education or vaccines. Implementation of influenza or other vaccine educational strategies in the ED can assist in decreasing vaccine-related illness and the burden of these diseases in the community during influenza season or a pandemic.

## Data Availability

All data generated or analyzed during this study are included in this published article (and its supplementary information files).
